# RPGR, a prenylated retinal ciliopathy protein, is targeted to cilia in a prenylation- and PDE6D-dependent manner

**DOI:** 10.1242/bio.020461

**Published:** 2016-08-04

**Authors:** Nirmal Dutta, Seongjin Seo

**Affiliations:** Department of Ophthalmology and Visual Sciences, University of Iowa College of Medicine, Iowa City, IA 52242, USA

**Keywords:** Retinitis pigmentosa, Primary cilia, Trafficking, Photoreceptor degeneration, Prenylation

## Abstract

RPGR (retinitis pigmentosa GTPase regulator) is a ciliary protein associated with several forms of inherited retinal degenerative diseases. PDE6D is a ubiquitously expressed prenyl-binding protein and involved in ciliary targeting of prenylated proteins. The current working model for the RPGR function depicts that RPGR acts as a scaffold protein to recruit cargo-loaded PDE6D to primary cilia. Here, we present evidence demonstrating an alternative relationship between RPGR and PDE6D, in which RPGR is a cargo of PDE6D for ciliary targeting. We found that the constitutive isoform of RPGR, which is prenylated, requires prenylation for its ciliary localization. We also found that there are at least two independent ciliary targeting signals in RPGR: one within the N-terminal region that contains the RCC1-like domain and the other near the prenylation site at the C-terminus. Ablation of PDE6D blocked ciliary targeting of RPGR. Our study indicates that prenylated RPGR is one of the cargos of PDE6D for ciliary trafficking and provides insight into the mechanisms by which RPGR is targeted to cilia.

## Abstract (precis)

**Summary:** RPGR is a ciliary protein that functions as a scaffold to recruit cargo-loaded PDE6D to cilia. Our study shows that RPGR is also a cargo of PDE6D.

## INTRODUCTION

Protein prenylation is a post-translational lipid modification that adds a farnesyl or geranylgeranyl moiety to the C-terminus of a protein. This post-translational modification mediates protein-membrane and protein-protein interactions, affecting target protein's localization and function. Several proteins (e.g. PDE6 α and β subunits, GRK1, transducin γ subunit, RPGR, and INPP5E) that localize to primary cilia and the photoreceptor outer segment, which is a modified cilium, are prenylated, and mutations in these genes cause photoreceptor degeneration (Huang et al., 1995; Bowes et al., 1990; Yamamoto et al., 1997; Vervoort et al., 2000; Meindl et al., 1996; Bielas et al., 2009; Jacoby et al., 2009; Roepman et al., 1996; McLaughlin et al., 1993). Furthermore, mutations in genes involved in protein prenylation or ciliary trafficking of prenylated proteins (e.g. *CHM*, *RCE1*, *ARL3*, *PDE6D*, *RP2*, and *ARL13B*) also cause retinal degenerations in vertebrates (Seabra et al., 1993; Christiansen et al., 2011; Hanke-Gogokhia et al., 2016; Wright et al., 2016; Thomas et al., 2014; Zhang et al., 2007, 2015; Schwahn et al., 1998; Cantagrel et al., 2008), underscoring the importance of protein prenylation and proper trafficking of prenylated proteins to cilia in photoreceptor function and survival. However, mechanisms by which prenylated proteins are targeted to cilia and the photoreceptor outer segment are not sufficiently understood.

RPGR is a ciliary protein associated with several forms of inherited retinal degenerative diseases (Vervoort et al., 2000; Meindl et al., 1996; Demirci et al., 2002; Shu et al., 2007). Mutations in *RPGR* account for 50-60% of all X-linked retinitis pigmentosa (RP) cases and up to 20% of all RP cases (Breuer et al., 2002; Churchill et al., 2013; Shu et al., 2007). In addition, certain RPGR mutations are involved in cone-rod dystrophy (CRD) (Demirci et al., 2002). Two major isoforms of RPGR are expressed in vertebrates: RPGR^ex1-19^ and RPGR^ORF15^ (Kirschner et al., 1999; Hong and Li, 2002). The RPGR^ex1-19^ isoform (hereafter RPGR) is constitutively expressed in multiple organs, including the eye, and geranylgeranylated at its C-terminus. This isoform localizes to primary cilia (Gakovic et al., 2011). The RPGR^ORF15^ isoform is specifically expressed in the retina and localizes to the connecting cilium of photoreceptors (Hong et al., 2003; Wright et al., 2011, 2012). Contrary to the constitutive isoform, RPGR^ORF15^ is not geranylgeranylated. How these RPGR isoforms are targeted to primary cilia and to the photoreceptor-connecting cilium is not well understood.

PDE6D (also known as PrBP/δ and PDEδ) is a ubiquitously expressed prenyl-binding protein (Florio et al., 1996; Zhang et al., 2004; Nancy et al., 2002). By binding to prenyl moieties, PDE6D is thought to mediate shuttling of prenylated proteins between membranes (Baehr, 2014; Schmick et al., 2014; Chandra et al., 2012). Recent studies found that PDE6D is involved in ciliary trafficking of INPP5E, a protein linked to Joubert syndrome (Humbert et al., 2012; Bielas et al., 2009; Jacoby et al., 2009; Thomas et al., 2014). PDE6D is also thought to be involved in the outer-segment targeting of PDE6 α/β subunits, transducin γ subunit, and GRK1 in photoreceptors because these proteins either partly mis-localize or are degraded in *Pde6d^−/−^* photoreceptors (Zhang et al., 2007).

Previous studies uncovered that RPGR binds to PDE6D through its N-terminal RCC1-like domain (RLD) (Linari et al., 1999; Wätzlich et al., 2013). Based on structural analyses, Wätzlich et al. further proposed that RPGR acts as a scaffold protein to recruit cargo-loaded PDE6D to primary cilia (Wätzlich et al., 2013). More recently, however, an alternative mode of interaction between prenylated RPGR and PDE6D was reported (Lee and Seo, 2015). This study found that PDE6D binds to the C-terminus of RPGR in a prenylation-dependent manner, suggesting that prenylated RPGR could be a cargo of PDE6D in addition to acting as a docking site for cargo-loaded PDE6D. Here, we address this question and report how the prenylated, constitutive isoform of RPGR is targeted to cilia.

## RESULTS

### Ciliary localization of RPGR is prenylation-dependent

**Fig. 1.**
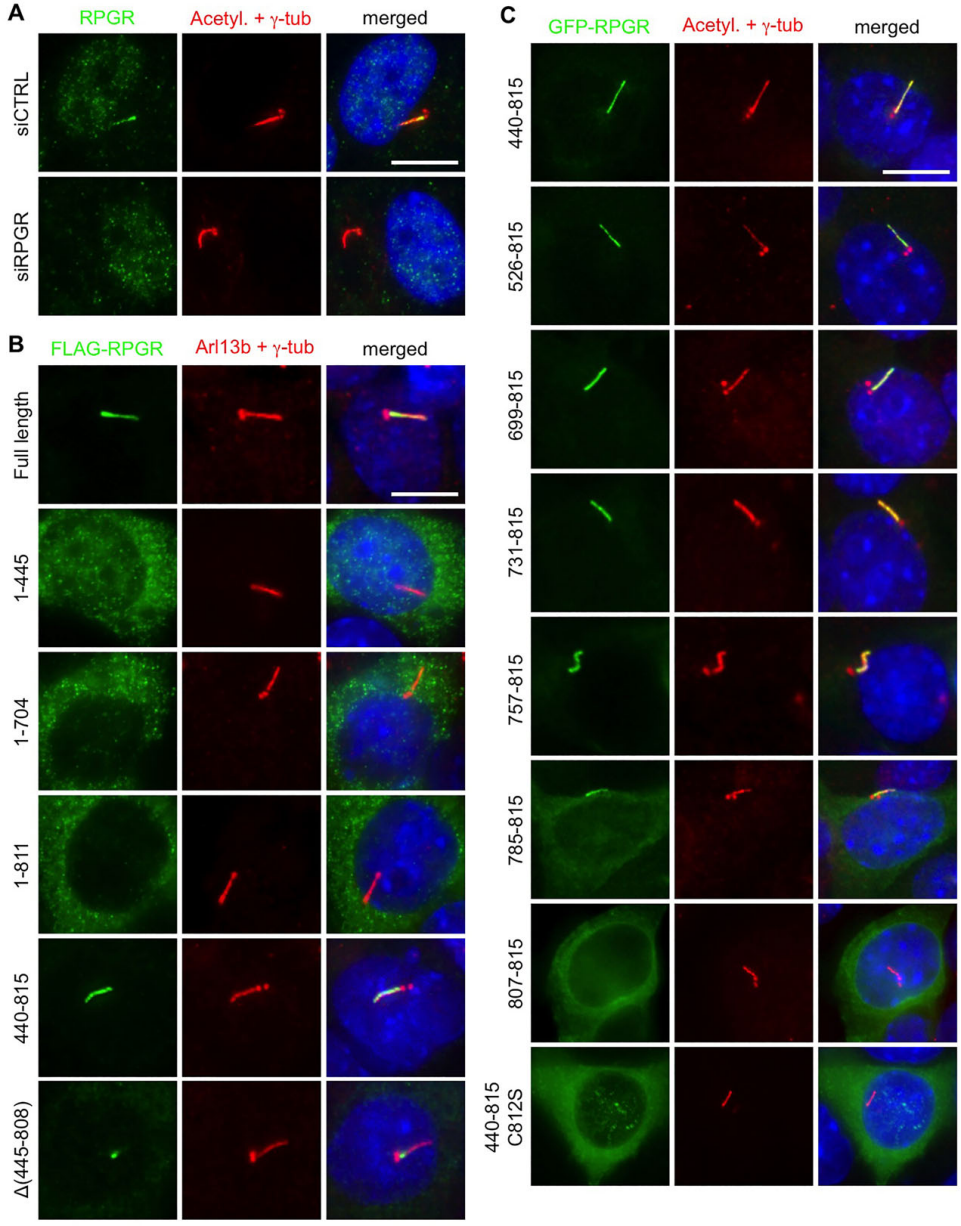
**Localization of RPGR and its mutant variants.** (A) Localization of endogenous RPGR in hTERT-RPE1 cells. hTERT-RPE1 cells were transfected with either non-targeting (siCTRL) or human RPGR (siRPGR) siRNAs and the localization of RPGR was probed with anti-RPGR antibodies (green). Cilia and centrosomes (red) were marked with anti-acetylated tubulin and anti-γ-tubulin antibodies. Nuclei were stained with DAPI (blue). (B) Localization of FLAG-tagged RPGR mutant variants in IMCD3 cells. IMCD3 cells were transiently transfected with indicated FLAG-RPGR constructs and localization of RPGR variants were probed with anti-FLAG antibodies (green). Cilia and centrosomes (red) were marked with anti-Arl13b and anti-γ-tubulin antibodies. Numbers denote the amino acid residues included in the construct, while Δ symbolizes a deletion. Nuclei were stained with DAPI (blue). (C) Localization of GFP-tagged RPGR mutant variants. IMCD3 cells were transfected with plasmids expressing indicated GFP-RPGR mutant variants and their localization was examined with anti-GFP antibodies (green). Anti-acetylated tubulin and anti-γ-tubulin antibodies were used to visualize cilia and centrosomes (red). Nuclei were stained with DAPI (blue). Scale bars: 10 µm.

RPGR is previously shown to localize to primary cilia (Gakovic et al., 2011). We validated this finding by immunohistochemistry against endogenous RPGR and knocking down *RPGR* expression using small interfering RNAs (siRNAs) (Fig. 1A). Although RPGR is detected throughout the cilium, it should be noted that RPGR is significantly enriched within the proximal region of the cilium, which encompasses the transition zone and the proximal end of the ciliary shaft. To dissect the mechanism of RPGR ciliary targeting and identify the minimal element(s) for that, we generated various RPGR deletion and substitution mutant variants with either a FLAG or a GFP tag. These constructs were transfected into IMCD3 cells and their localization was examined (Fig. 1B,C). Consistent with the ciliary localization of endogenous RPGR, FLAG-tagged full-length RPGR (FLAG-RPGR; aa 1-815) showed specific localization to cilia in IMCD3 cells. Similar to endogenous RPGR, FLAG-RPGR exhibited enrichment within the proximal region of the cilium, particularly in low-expressing cells. Then we examined the localization of RPGR deletion mutants. FLAG-RPGR 1-445, 1-704, and 1-811 all showed cytoplasmic localization, while the C-terminal half of RPGR (aa 440-815) exhibited specific ciliary localization but without enrichment near the ciliary base. These data suggest that prenylation of RPGR at the C-terminus may be essential for its ciliary localization and that the C-terminal half of RPGR is sufficient to target it to cilia. To test the requirement of prenylation for RPGR ciliary targeting, we generated a prenylation-incompetent mutant, in which the Cys residue within the CaaX prenylation motif was mutated to Ser (C812S), and examined its localization. As shown in Fig. 1C, this mutation abrogated ciliary localization of RPGR. Therefore, we conclude that prenylation is essential for RPGR ciliary targeting.

Recently, high affinity binding of PDE6D to prenylated cargos combined with specific release by a ciliary small GTPase ARL3 was suggested as a determinant for ciliary targeting of prenylated proteins (Fansa et al., 2016). Since geranylgeranylated RPGR is predicted to bind to PDE6D with a low-nanomolar range equilibrium dissociation constant (K_d_), which is similar to a high-affinity ciliary cargo INPP5E (Fansa et al., 2016, 2015), we tested whether prenylation and high-affinity binding to PDE6D is sufficient for ciliary targeting. To this end, we examined the localization of GFP fused with the last nine amino acids of RPGR (aa 807-815), which is sufficient to bind to PDE6D (Lee and Seo, 2015). As shown in Fig. 1C, GFP-RPGR 807-815 failed to localize to cilia, suggesting that prenylation and high affinity binding to PDE6D is not sufficient for ciliary targeting and that ciliary targeting or retention signals are needed.

### Ciliary-targeting signals in RPGR

We then sought to determine additional elements that target RPGR to cilia. While the N-terminal half of RPGR (aa 1-445) failed to localize to cilia, the C-terminal half was sufficient, implying that the ciliary targeting signal(s) are within the latter half. This finding was interesting because it was previously shown that RPGRIP1 was necessary for the connecting cilium localization of RPGR in photoreceptors and that RPGR bound to RPGRIP1 and its structural homolog RPGRIP1L through the RLD (Zhao et al., 2003; Roepman et al., 2000; Boylan and Wright, 2000; Patil et al., 2012; Khanna et al., 2009). To test whether the N-terminal half of RPGR has any role in ciliary localization of RPGR and whether the sequences in the C-terminal half (except for the prenylation motif) are essential for RPGR ciliary targeting, we generated an internal deletion mutant, in which residues between aa 444 and 809 were removed [Δ(445-808)]. It should be noted that the Ser residue at the -3 position (Ser809) relative to the CaaX motif (C is at the 0 position), which is necessary for PDE6D binding (Lee and Seo, 2015), is preserved in this mutant. Interestingly, unlike the full-length RPGR, this deletion mutant [RPGR Δ(445-808)] specifically localized to the ciliary transition zone but was not detected in more distal parts of the cilium (Fig. 1B). In contrast, RPGR 440-815 mutants, which lacked the RLD, were found evenly distributed throughout the cilium with no enrichment at the ciliary base (Fig. 1B,C). These findings suggest that the N-terminal RLD is sufficient to target RPGR to the transition zone as long as RPGR is prenylated (or membrane-anchored). These data also suggest that the RLD in RPGR, presumably by binding to RPGRIP1 and RPGRIP1L (Roepman et al., 2000; Boylan and Wright, 2000; Khanna et al., 2009), induces enrichment of RPGR within the proximal region of the cilium.

To map the ciliary targeting signal(s) within the C-terminal portion of RPGR, we generated a series of deletion mutants with an N-terminal GFP tag (aa 526-815, 699-815, 731-815, 757-815, and 785-815). GFP-RPGR 526-815, 699-815, 731-815, and 757-815 all showed specific localization to cilia (Fig. 1C). GFP-RPGR 785-815 was able to localize to cilia but a considerable amount was found mis-localized to the cytoplasm, indicating that the ciliary targeting signal is partly but not completely compromised. As mentioned above, GFP-RPGR 807-815 was found dispersed throughout the cell. These data indicate that the C-terminal ciliary targeting signal(s) are located between aa 757 and 806. In summary, our data show that RPGR has at least two independent ciliary targeting signals: one within the RLD and the other near the C-terminus.

### PDE6D is essential for ciliary targeting of RPGR

**Fig. 2.**
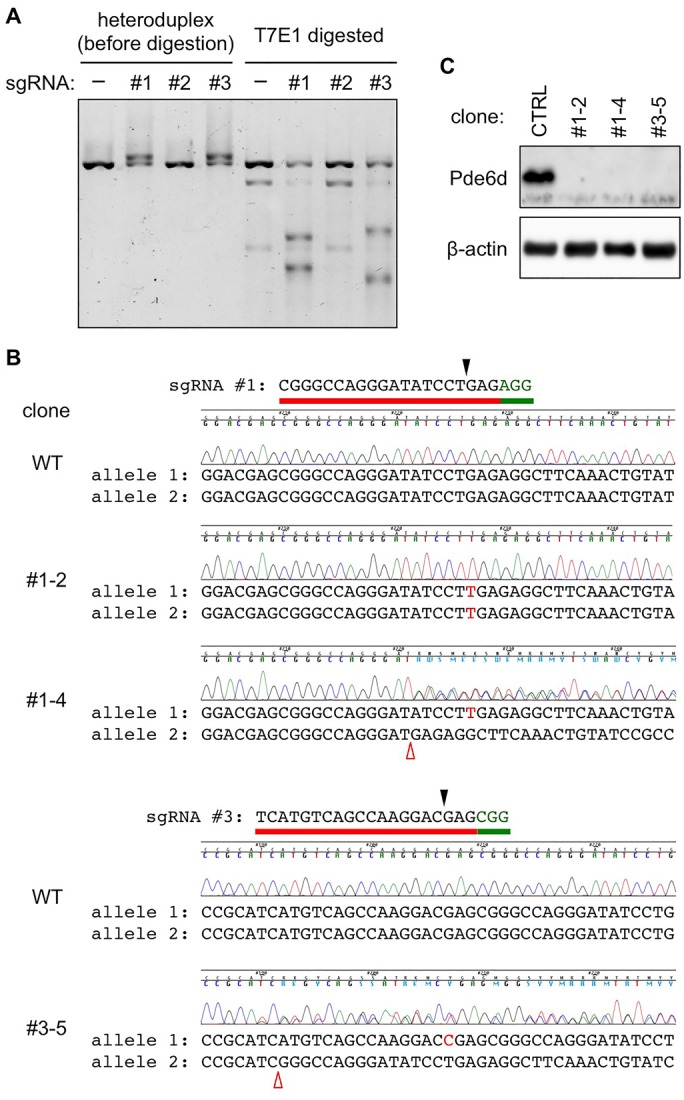
**Generation of *Pde6d* knockout cells.** (A) Effective genome editing at *Pde6d* exon 1 by sgRNAs #1 and #3 in IMCD3 cells. Genomic DNAs were extracted from normal or indicated sgRNA transfected IMCD3 cells and DNA fragments containing *Pde6d* exon 1 were amplified by PCR (see Materials and Methods for more detail). Heteroduplex DNAs before and after T7 endonuclease I (T7E1) digestion were visualized on a 2% agarose gel. (B) Sequencing results of the target region in normal (WT, wild-type) and selected *Pde6d* null mutant cell lines. Target site sequences for sgRNAs #1 and #3 are shown above the WT sequence chromatogram. Red bars indicate the sgRNA target sequences, and green bars indicate the protospacer adjacent motif (PAM). Black arrowheads mark the expected double stranded break (DSB) sites. Red open arrowheads denote deletions and inserted nucleotides are written in red upper case letters. Both alleles in clone #1-2 have identical 1-nucleotide (nt) insertions. Alleles in clone #1-4 have a 1-nt insertion and a 5-nt insertion. Alleles in clone #3-5 have a 1-nt insertion and a 19-nt deletion. (C) Immunoblotting against Pde6d verifies the lack of Pde6d proteins in knockout cells. Protein extracts were prepared from the indicated clonal cell lines and the quantity of Pde6d proteins was assessed by immunoblotting. β-actin was used as a loading control.

Given the requirement of prenylation for RPGR ciliary targeting, we sought to determine the role of PDE6D in the same process. To this end, we generated *Pde6d* null mutant cell lines using the CRISPR (clustered regularly interspaced short palindromic repeats)/Cas9 technology and examined RPGR localization. We designed and tested three single guide RNAs (sgRNAs) targeting mouse *Pde6d* exons 1 and 2 (Fig. 2; also see Materials and Methods for sgRNA sequences and vector construction). After transient transfection into IMCD3 cells, sgRNAs #1 and #3 effectively induced indels as determined by the T7E1 assay (Fig. 2A). Therefore, we used these two sgRNAs to generate *Pde6d* null mutant cell lines. Following sequence analyses of the *Pde6d* locus in individual clones, a total of three independent *Pde6d* null mutant lines were chosen for further studies (clones #1-2, #1-4, and #3-5; Fig. 2B). The absence of Pde6d proteins in these cell lines was verified by immunoblotting (Fig. 2C).

**Fig. 3.**
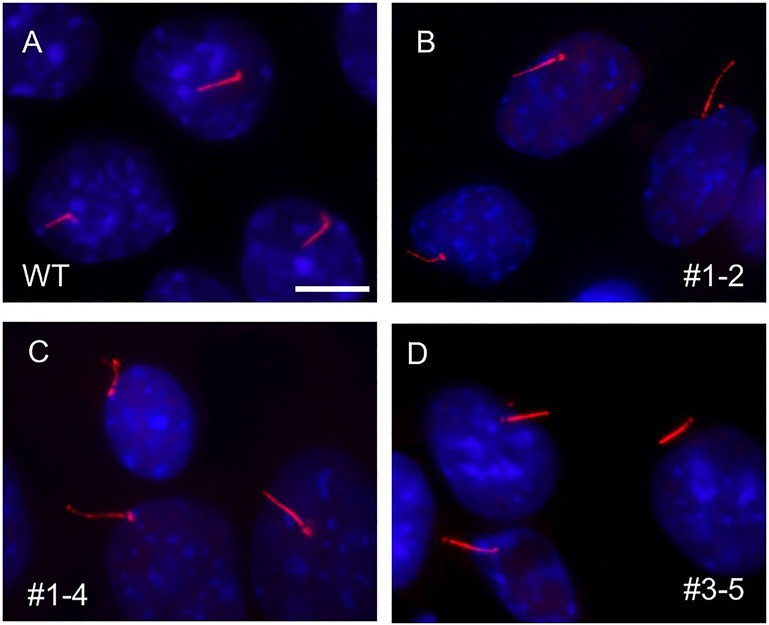
**Normal ciliogenesis in the absence of Pde6d.** Cilia formation was examined by indirect immunofluorescence microscopy in (A) normal (WT, wild type) and (B-D) *Pde6d* null (clones #1-2, #1-4, and #3-5) IMCD3 cell lines. Cells were serum-starved for 48 h and the presence of primary cilium was examined by anti-acetylated tubulin and anti-γ-tubulin antibodies (red). Nuclei were stained with DAPI (blue). Scale bar: 10 µm.

We first examined whether ciliogenesis is altered by the loss of Pde6d. Normal and *Pde6d* null mutant IMCD3 cells were cultured in a serum-free medium to induce ciliogenesis and cilium formation was examined by immunocytochemistry using anti-acetylated tubulin and anti-γ-tubulin antibodies. Consistent with the previous observation in human fibroblasts derived from a Joubert syndrome patient with a 42-amino acid in-frame deletion (Thomas et al., 2014) and the relatively normal development of the photoreceptor outer segment in *Pde6d^−/−^* mice (Zhang et al., 2007), complete loss of Pde6d function did not cause any ciliogenesis defect (Fig. 3; *n*>100 in each cell line). These data indicate that Pde6d is not required for ciliogenesis.

**Fig. 4.**
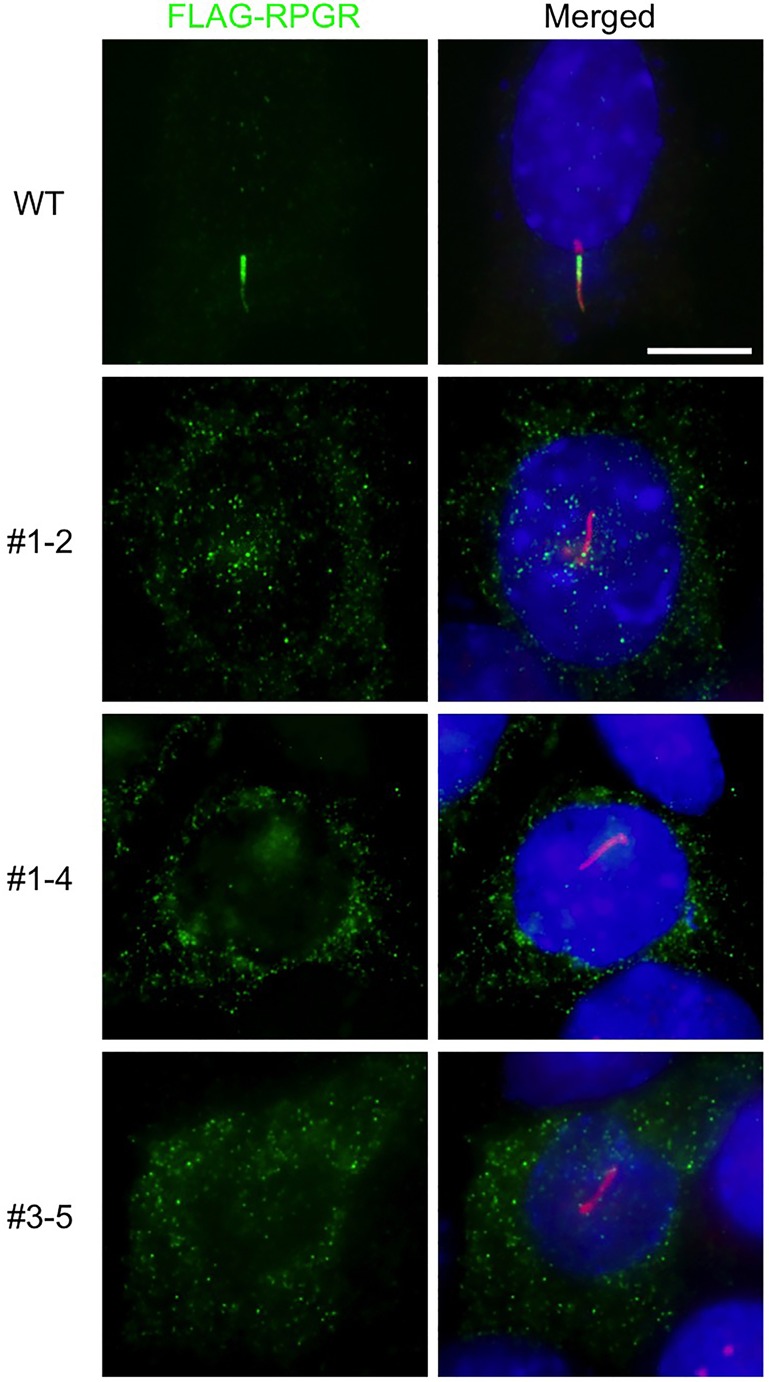
**Pde6d is essential for RPGR ciliary targeting.** FLAG-RPGR (full-length) was transiently transfected into normal and indicated Pde6d null mutant cell lines and its localization was probed with anti-FLAG antibodies (green). Cilia and centrosomes were marked with anti-Arl13b and anti-γ-tubulin antibodies (red). Nuclei were stained with DAPI (blue). Scale bar: 10 µm.

We then investigated the requirement of Pde6d in RPGR ciliary targeting; to this end, we transfected FLAG-RPGR into normal and *Pde6d* null IMCD3 cells. As shown in Fig. 4, ablation of Pde6d completely abolished RPGR ciliary localization in all three *Pde6d* null mutant cell lines; no ciliary localization was observed in more than 50 transfected cells in each mutant cell line. Therefore, we conclude that Pde6d is essential for RPGR ciliary targeting.

## DISCUSSION

Our study reveals three key findings with respect to the mechanisms of RPGR ciliary targeting: (i) RPGR has at least two independent ciliary targeting signals, (ii) prenylation is essential for RPGR ciliary targeting, and (iii) prenylated RPGR is a cargo of PDE6D. As mentioned in the Introduction, RPGR interacts with PDE6D in two distinct modes (Wätzlich et al., 2013; Linari et al., 1999; Lee and Seo, 2015; Fansa et al., 2015). One of these modes is through its RLD and the other is through its C-terminal prenylation site. Through the RLD-mediated interaction, RPGR was proposed to act as a docking site to recruit prenylated cargo-PDE6D complexes to cilia (Wätzlich et al., 2013; Baehr, 2014). Our study uncovers the significance of the prenylation-dependent interaction between RPGR and PDE6D and demonstrates that prenylated RPGR is a cargo of PDE6D. Our study further suggests that the RLD in RPGR has an additional role in RPGR ciliary localization; it limits or enriches RPGR to the transition zone and the proximal end of the cilium. RPGR binds to RPGRIP1 and RPGRIP1L, which specifically localize to the transition zone in primary cilia and the connecting cilium in photoreceptors, through the RLD (Roepman et al., 2000; Boylan and Wright, 2000; Khanna et al., 2009). Furthermore, Rpgr was found mis-localized in *Rpgrip1* knockout photoreceptors (Patil et al., 2012; Zhao et al., 2003). Therefore, we predict that the enrichment of RPGR at the transition zone and the proximal region of the cilium is dependent on the RLD-RPGRIP1/RPGRIP1L interactions.

**Fig. 5.**
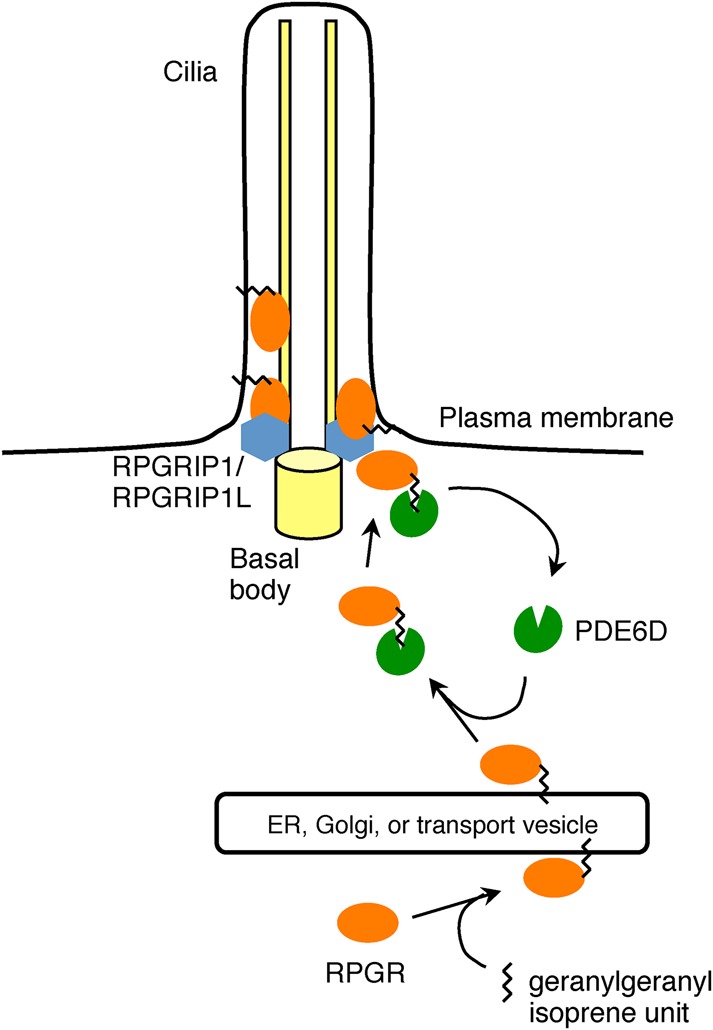
**A schematic model for RPGR ciliary targeting.**

Based on our findings and previous studies (Baehr, 2014; Wätzlich et al., 2013; Ismail et al., 2011; Zhao et al., 2003; Roepman et al., 2000; Boylan and Wright, 2000; Patil et al., 2012; Khanna et al., 2009), we propose a model for RPGR ciliary targeting (Fig. 5). The constitutive isoform of RPGR is geranylgeranylated upon its synthesis and anchored onto an endomembrane through its geranylgeranyl moiety. The identity of this endomembrane is currently unknown but candidates are endoplasmic reticulum, Golgi, and endosomal membranes. PDE6D is required for the extraction of RPGR from the donor membrane and shuttles it to the plasma membrane of the primary cilium. Although not validated yet, GTP-bound ARL3, a small GTPase that localizes to cilia, is expected to bind to PDE6D and induce the release of RPGR from PDE6D and eventually RPGR membrane anchoring. Interactions with RPGRIP1 and RPGRIP1L in the transition zone prevent RPGR diffusion and limit its localization to the transition zone and the proximal end of the primary cilium. RPGRIP1 and RPGRIP1L may also be involved in the initial entry of the RPGR-PDE6D complex into the cilium.

It should be noted that the prenylation- and PDE6D-dependent ciliary targeting of RPGR is limited to the constitutive isoform of RPGR and that the photoreceptor-specific RPGR^ORF15^ isoform may be targeted to the photoreceptor connecting cilium through a distinct mechanism. This is because RPGR^ORF15^ does not have the CaaX prenylation motif and therefore it is expected not to interact with PDE6D through its C-terminus. Instead, RPGR^ORF15^ has a Glu-rich domain at its C-terminus. Although the function of the Glu-rich domain is currently not well understood, recent studies found that it is glutamylated by tubulin tyrosine ligase like-5 (TTLL5) in photoreceptors (Sun et al., 2016; Rao et al., 2016). Since the RLD has a role in ciliary localization of RPGR but it is not sufficient for its ciliary targeting on its own (i.e. without prenylation), it is tempting to speculate that the Glu-rich domain cooperates with the RLD and contributes to the connecting cilium localization of RPGR^ORF15^ in photoreceptors.

In summary, our study demonstrates that the constitutive isoform of RPGR is targeted to cilia in a prenylation- and PDE6D-dependent manner and, therefore RPGR is a cargo of PDE6D.

## MATERIALS AND METHODS

### Reagents

Antibodies used in this study and their dilutions for immunocytochemistry are as follows: rabbit polyclonal anti-RPGR (Sigma #HPA001593; 1:100 dilution), mouse monoclonal anti-acetylated tubulin (Proteintech group #66200-1-Ig; 1:2500), mouse monoclonal anti-γ-tubulin (Sigma #T6557; 1:2500), rabbit polyclonal anti-γ-tubulin (Sigma #T3559; 1:2500), mouse monoclonal anti-FLAG (Sigma #F1804; 1:2000), rabbit polyclonal anti-ARL13B (Proteintech group #17711; 1:2000), rabbit monoclonal anti-GFP (Invitrogen #G10362; 1:500), rabbit polyclonal anti-PDE6D (MyBiosources #MBS7005086; 1:1000 for immunoblotting), mouse monoclonal anti-β-actin (Sigma #A1978; 1:10,000 for immunoblotting). Small interfering RNAs (siRNAs) for control (ON-TARGETplus non-targeting siRNA) and human RPGR (ON-TARGETplus SMARTpool) were obtained from GE Dharmacon.

### Constructs

The open reading frame encoding full-length RPGR^ex1-19^ was obtained by PCR using the Human Retina Marathon-ready cDNA library (Clontech) and the Expand High Fidelity PCR system (Roche) and cloned into pSS-FS plasmid (Humbert et al., 2012) for N-terminal FLAG tag fusion. For N-terminal GFP fusion, pEGFP-C3 (Clontech) plasmid was used. Deletion and substitution mutagenesis was conducted by PCR and standard molecular biology methodologies. All constructs were sequence-verified.

### Immunocytochemistry

Mouse IMCD3 (CRL-2123) and human hTERT-RPE1 (CRL-4000) cells were obtained from American Type Culture Collection (ATCC) and cultured in DMEM/F12 (Invitrogen) supplemented with 10% fetal bovine serum (Invitrogen) and penicillin/streptomycin (Invitrogen). Transient transfection of plasmid DNAs was conducted with FuGene HD (Promega) and siRNAs were transfected with RNAiMAX (Invitrogen). Cells were seeded on glass coverslips in 24-well plates and transfected with indicated DNAs or siRNAs following the manufacturer's instruction. After 24-48 h of transfection, cells were incubated in a serum-free medium for 24-36 h to induce ciliogenesis. For immunocytochemistry, cells were fixed in 4% paraformaldehyde (PFA) in PBS followed by cold methanol. Samples were blocked with 5% bovine serum albumin (BSA) and 2% normal goat serum in PBST (PBS+0.1% Triton X-100) and incubated with indicated primary antibodies in the blocking buffer for 2 h at room temperature. After washing in PBST, coverslips were incubated with goat anti-mouse or goat anti-rabbit secondary antibodies conjugated with Alexa Fluor 488 and 568 (Invitrogen). Coverslips were mounted on VectaShield mounting medium with DAPI (Vector laboratories) and images were taken with Olympus IX71 microscope.

### Generation of Pde6d knockout cells

*Pde6d* knockout IMCD3 cells were generated by using the CRISPR/Cas9 technology (Ran et al., 2013). Three sgRNAs targeting mouse *Pde6d* exons 1 and 2 were designed using a publicly available sgRNA designer tool (http://www.broadinstitute.org/rnai/public/analysis-tools/sgrna-design) (Doench et al., 2016) and cloned into the pX459 plasmid (Ran et al., 2013) (Addgene). Oligonucleotide sequences for sgRNA cloning are as follows: sgRNA #1; 5′-CACCGCGGGCCAGGGATATCCTGAG-3′ and 5′-AAACCTCAGGATATCCCTGGCCCGC-3′, sgRNA #2; 5′-CACCGACCTTCGGGATGCCGAAACA-3′ and 5′-AAACTGTTTCGGCATCCCGAAGGTC-3′, sgRNA #3; 5′-CACCGTCATGTCAGCCAAGGACGAG-3′ and 5′-AAACCTCGTCCTTGGCTGACATGAC-3′. For the initial sgRNA screening, pX459 plasmids with these sgRNAs were transiently transfected into IMCD3 cells for 24 h, and transfected cells were selected by puromycin treatment (0.25 µg/ml) for 2 days. Following expansion in normal growth media, cells were harvested for genomic DNA extraction and *Pde6d* exons 1 and 2 were PCR-amplified using the following primers and the Universe Hot Start High-Fidelity DNA polymerase (Biotool): for exon 1; 5′-GCTGGCTTGTCATTGGTTTC-3′ and 5′-GGCCTTCAGGACCCTAATTT-3′, for exon 2; 5′-GTTCCAGGACAGGGATTGTT-3′ and 5′-TGGGAAGTGAGAGAAGGAAATG-3′. For T7E1 assays, heteroduplex DNAs were generated by denaturing PCR products at 95°C for 5 min and slowly cooling down to room temperature. Heteroduplex DNAs were digested with T7 endonuclease I (NEB) for 30 min at 37°C. Digested DNAs were loaded on a 2% agarose gel to assess genome editing efficiencies. To generate *Pde6d* knockout clonal cell lines, IMCD3 cells were transfected with pX459 plasmids with sgRNAs #1 and #3, selected with puromycin as above, and seeded at a low density in 10-cm tissue culture dishes. After 2 weeks of culture, well-separated individual clones were trypsinized and transferred into 24-well plates and expanded. Genomic DNAs were extracted from a subset of cells, and *Pde6d* exon 1 was PCR amplified using the primers described above and sequenced. Clones with frame-shift mutations were selected and the lack of Pde6d proteins was verified by immunoblotting.
